# Aminophosphonates in Nanofiltration and Reverse Osmosis Permeates

**DOI:** 10.3390/membranes11060446

**Published:** 2021-06-15

**Authors:** Ramona Kuhn, Carsten Vornholt, Volker Preuß, Isaac Mbir Bryant, Marion Martienssen

**Affiliations:** 1 Chair of Biotechnology of Water Treatment, Brandenburg University of Technology Cottbus/Senftenberg, 03046 Cottbus, Germany; martiens@b-tu.de; 2 Chair of Water Treatment and Urban Hydraulic Engineering, Brandenburg University of Technology Cottbus/Senftenberg, 03046 Cottbus, Germany; carstenvornholt@gmx.de (C.V.); preuss@b-tu.de (V.P.); 3 Department of Environmental Science, University of Cape Coast, Cape Coast 4P4872, Ghana; ibryant@ucc.edu.gh

**Keywords:** ATMP, AMPA, phosphonates, nanofiltration, reverse osmosis, antiscalants, drinking water treatment

## Abstract

Aminophosphonates such as aminotris(methylenephosphonic acid) (ATMP) are common constituents of antiscalants. In nanofiltration (NF) and reverse osmosis (RO) processes, ATMP prevents inorganic scaling leading to more stable membrane performance. So far, little attention has been paid to the possible permeation of aminophosphonates through NF and RO membranes. We have investigated the permeability of these membrane types for ATMP and its potential metabolites iminodi(methylenephosphonic acid) (IDMP) and amino(methylenephosphonic acid) (AMPA) with two different NF membranes (TS40 and TS80) and one RO membrane (ACM2) and three different water compositions (ultra-pure water, synthetic tap water and local tap water). We found traces of phosphonates in all investigated permeates. The highest phosphonate rejection occurred with local tap water for all three membranes investigated. Filtration experiments with a technical antiscalant formulation containing ATMP indicated similar trends of phosphonate permeability through all three membranes. We assume that the separation mechanisms of the membranes are the results of a very complex relationship between physico-chemical properties such as Donnan exclusion, feed pH, feed ionic strength and feed concentration, as well as solute–solute interactions.

## 1. Introduction

Nanofiltration (NF) and reverse osmosis (RO) processes are well-established applications in softening water and desalination for drinking water production [1]. Their high process stability is an important prerequisite for ensuring sufficient efficiency. This can be negatively influenced by the supersaturation of poorly soluble salts of a divalent cation, such as calcium (Ca^2+^) and/or magnesium (Mg^2+^), resulting in membrane blockage and declining permeate yields. The lifetime and performance of the membrane can drastically shorten if scaling remains disregarded due to membrane interruptions. In order to reduce scaling effects, antiscaling agents (also termed antiscalants) are commonly applied. In desalination of brackish water RO for drinking water production, the use of antiscalants is of major importance and increases the water flux recovery and decreases the operating cost making this process more efficient [2,3]. Antiscalants comprise a group of synthetic organic polymers such as polyacrylic acids, carboxylic acids, polymaleic acids, polyphosphates, anionic polymers and phosphonates [4]. The latter, such as aminotris(methylenephosphonic acid) (ATMP), is well-known to prevent the scaling of calcium salts at the early stages of the crystallisation process. Therefore, phosphonates can be dosed at substoichiometric ratios to earth alkali ions [5]. As a result, the required concentration of antiscalants using phosphonates is often lower compared to other antiscalants.

Nevertheless, commercial phosphonate-based antiscalants can contain significant portions of impurities. Armbruster et al. [6] found up to 20% phosphorous impurities in technical ATMP formulations. They identified at least six different by-products and pointed out that these compounds might also be present in the permeates. One major impurity was amino(methylenephosphonic acid) (AMPA). The smallest aminophosphonate is also better known as the major metabolite of the most famous aminophosphonate glyphosate. However, AMPA can also be a by-product of antiscalant ATMP. In Germany, some drinking water applications using NF recently detected traces of AMPA in drinking water permeates as predicted by Armbruster et al. [6].

The presence of traces of AMPA in drinking water permeates is a big issue because AMPA is suspected to be the cause of health risks to humans even at a low mass fraction [7,8]. In 2003, the world health organization (WHO) clearly stated that the presence of AMPA in drinking water does not represent a hazard to human health [9]. Grandcoin et al. [8] summarized the most relevant effects on human health caused by AMPA in their review. In particular, Kwiatkowska et al. [10] showed that 0.05 mM AMPA can have slightly toxic effects on human erythrocytes and can induce hemolysis. Others reported that 1.8 mM AMPA can have clastogenic effects [11] and might cause umbilical membrane cell damages [12].

With regard to the current debate on the utilization of glyphosate in agriculture and its potential to degrade to AMPA, some scientists pointed out that there is not always a direct correlation between AMPA exposure and the use of glyphosate [13,14]. In particular, Hoppe [14] showed that the determined ratios of AMPA/glyphosate could be very variable, especially in human urine samples. The author proposed that in those cases where no direct correlation between AMPA and glyphosate occurs, additional sources of aminophosphonates, such as ATMP or ethylenediaminetetra(methylenephosphonic acid) (EDTMP), might be considered because those can also degrade to AMPA. In drinking water production, the occurrence of AMPA was recently reported for different sources such as groundwater [15], agriculture watersheds [16] and streams [17]. So far, the drinking production process itself was not considered as an additional source of aminophosphonates such as ATMP and AMPA.

Legal regulations in Germany on drinking water production and its quality have to fulfil high standards. According to the German drinking water ordinance for human consumption (§ 11 Treatment materials and disinfection methods), the use of aminophosphonates such as ATMP is permitted for water treatment. However, the observation of potential metabolites in drinking water permeates requires more detailed investigations on the membrane process itself. It is still unclear whether the presence of AMPA in those drinking water permeates observed is a result of overloading the membrane concentrate by technical impurities of phosphonate-based antiscalants or by physico-chemical and/or biological processes breaking down the mother compounds.

Overall, the chemical behaviour of phosphonate-based antiscalants such as ATMP during the filtration process in NF and/or RO is poorly understood. Up to now, no attention has been paid to the fact that the antiscalants might also pass the membranes because it was assumed that phosphonates are retained in the concentrated brine. Thus, it is still uncertain whether the rejection behaviour of NF and RO also has an influence on the rejection of phosphonate-based antiscalants. It is well known that the separation mechanisms on NF membranes are a result of steric hindrance through the pore size, Donnan exclusion and dielectric exclusion causing charges on/in the membrane layer [18]. The separation mechanisms of RO are achieved by applying a semi-permeable membrane rejecting molecules by size exclusion, charge exclusion and physico-chemical interactions in the concentrate [19]. Furthermore, the ion rejection by NF and RO also depends on the operating conditions in terms of permeability coefficients, the feed space geometries and others [20].

Since it was assumed that phosphonates such as ATMP do not permeate through the membrane, there is currently no detailed information on whether those mechanisms might play an important role in the potential rejection of ATMP and smaller aminophosphonates. For that reason, in this study, we basically investigated the permeation of ATMP and its two metabolites, iminodi(methylenephosphonic acid) (IDMP) and AMPA, during NF and RO membranes using NF and RO membranes. Three different water compositions were tested, i.e., ultra-pure water, synthetic tap water and local tap water. Permeates were analysed for total phosphorus and aminophosphonates by LC/MS.

## 2. Materials and Methods

### 2.1. Chemicals and Reagents

**Figure 1:**
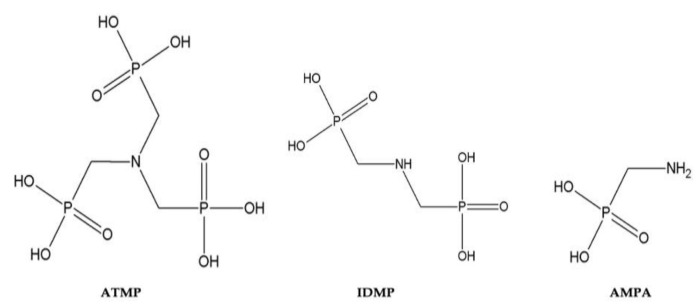
Chemical structures of ATMP, IDMP and AMPA.

ATMP was provided by “Zschimmer & Schwarz Mohsdorf” (Burgstädt, Germany). AMPA and IDMP and the internal standard glyphosate were purchased from Sigma-Aldrich (Steinheim, Germany). All phosphonates were of analytical grade or better, with purity >99%. The chemical structure of ATMP (299 g mol^−1^), IDMP (205 g mol^−1^) and AMPA (g mol^−1^) is presented below (Figure 1). Ultra-pure water (LC/MS grade) was in-house generated (Adrona Sia Crystal EX, Lithuania). Acetonitrile (LC/MS grade) was purchased from VWR (Darmstadt, Germany), and ammonium acetate (CH_3_COONH_4_; analytical grade) was purchased from VWR (Leuven, Belgium). Magnesium sulphate heptahydrate (MgSO_4_ × 7 H_2_O) was purchased from Merck (Darmstadt, Germany), and calcium carbonate (CaCO_3_) was obtained from Fluka (Seelze, Germany). Acetic acid was purchased from Riedel-de Haen (Seelze, Germany). The ICP standard solution IV (23 elements in 2% nitric acid—1000 mg L^−1^) was purchased from Roth (Karlsruhe, Germany). The multicomponent standard II for anions was purchased from Merck (Darmstadt, Germany).

### 2.2. Standard Solutions and Water Compositions

All membrane filtration experiments were carried out with ATMP, IDMP or AMPA at a concentration of 10 mg L^−1^. All solutions were always freshly prepared either with ultra-pure water (UPW), synthetic tap water or with local tap water (Cottbus, Germany). UPW was used to investigate only the interaction of the three different aminophopsphonates with the three selected membranes. The synthetic tap water composition was used to determine the specific influence of bivalent ions, namely calcium and magnesium. Finally, real tap water with a comparable higher water hardness caused by high calcium ion concentration was used to simulate a real water purification treatment.

The synthetic tap water was composed of 120 mg L^−1^ calcium carbonate (corresponding to 48 mgCa^2+^ L^−1^) and 100 mg L^−1^ magnesium sulphate (corresponding to 9.8 mgMg^2+^ L^−1^ and 39 mgSO_4_^2−^ L^−1^). The pH of the synthetic tap water was adjusted to 6.5 using acetic acid. The conductivity averaged 250 µS cm^−1^. The local tap water had a corresponding Ca^2+^ and Mg^2+^ concentration of 90 mg L^−1^ and 11 mg L^−1^, respectively. The sulphate concentration averaged 123 mgSO_4_^2−^ L^−1^. The pH was 7.5, and the conductivity of the tap water averaged 500 µS cm^−1^.

### 2.3. Membranes and Filtration Experiments

The filtration experiments were carried out with three different membranes purchased from Microdyn-Nadir (Wiesbaden, Germany). Two NF membranes (Trisep^®^ TS40 and TS80) and one RO membrane (Trisep^®^ ACM2) were used. The membrane specifications according to the manufacture information package are summarized in Table 1. The filtration experiments were carried out with the lab scale plant LSta80-2 from Sima-tec (Schwalmtal, Germany; Figures S1 and S2). The process water was pumped from a storage tank (7.5 L) to a flat sheet membrane module operating similar to a spiral-wound membrane system (according to manufacture information Sim-tec). All membranes were operated in cross-flow mode. Both permeate and feed (also called concentrate) were fed back to the storage tank. The conductivity and flow rate were determined at intervals of 1 min. The operational temperature was set to 20 °C. The NF membranes TS40 and TS80 were operated at 9 bar and the RO membrane ACM2 at 15 bar. The operating flux for the NF membrane TS40 was 52 L h^−1^m^−2^, while 39 L h^−1^m^−2^ and 41 L h^−1^m^−2^ were used with NF membrane TS80 and RO membrane ACM2, respectively.

Pressure and temperature stabilisation required 30 min to achieve the setting. Another 30 min was required to flush the sampling ports and the permeate recirculation line. Flushing feed was discharged. The sample ports were always flushed before sampling. The flushing permeate was recirculated back to the feed storage tank. The final sampling volume was 125 mL, which was reduced to 12.5 mL by evaporation (see Section 2.4) for total phosphorus measurements. For LC/MS analyses, 250 mL was taken and reduced to 5 mL by evaporation. The recovery rate of the NF membrane TS40 and TS80 averaged 0.6% and 0.9%, respectively. The recovery rate of the RO membrane ACM2 averaged 0.7%. Samples of permeates were taken after 1 h, 2 h and 3 h of the operation, representing technical triplicates.

Filtration experiments with a commercial antiscalant containing ATMP were carried out with local tap water and the same filtration setting as described above. This technical solution was diluted at 1:5000, resulting in a final concentration of 200 µL L^−1^ corresponding to a resulting phosphorus concentration of 12 mgP L^−1^.

### 2.4. Analytical Methods

Prior to analyses, all samples were concentrated with an evaporation system XcelVap (Horizon Technology, Salem, MA, USA). All analytical procedures were performed in plastic tubes to avoid strong phosphonate adsorption. The samples were resuspended in the smallest volume possible for further analysis. Phosphonates were analysed by liquid chromatography-electrospray ionization-mass spectrometry (LC-ESI-MS) using a Finnigan MAT LC/MS (LC spectral system P4000, LCQ MS Detector, autosampler AS 3000, Metal PEEK-coated column LUNA HILIC 100 × 2.0 mm, 3.0 µm/200 Å, Aschaffenburg, Germany). All liquid samples were mixed with 50% acetonitrile (VWR, Darmstadt, Germany) before injection. Gradient elution was performed with solvent A (100% ultra-pure water) and solvent B (10% ultra-pure water/90% acetonitrile) at 35 °C at a flow rate of 0.2 mL min^−1^. Both solvents A and B contained 2.5 mM ammonium acetate. The analysis was run for 43 min by first holding 100% of solvent B for 2 min. The gradient was then concavely increased to 10% A within 1 min and was held for 2 min. The gradient was further concavely increased to 30% A within 1 min and held again for 2 min. Subsequently, the gradient was again concavely increased to 50% A within 2 min and held for 10 min. Afterwards, the gradient was concavely increased to 60% A within 5 min and held for another 5 min, before the gradient was decreased back to 100% B within 3 min and held for 10 min before starting the next experiment. The MS detector settings were as follows: Negative polarity ionization at 3.5 kV, spray capillary temperature 220 °C. Selected ion monitoring (SIM) was chosen for quantification. The following mass-to-charge (m/z) ratios were used for identification: ATMP 298, IDMP 204, glyphosate 168 and AMPA 110. Glyphosate was used as a relative internal standard since phosphonate-based internal standards were not available.

Total phosphorous (TP) was determined after chemical digestion. Therefore, 200 mg of “Oxisolv” (Merck, Darmstadt, Germany) was added into a 5 mL sample volume. The samples were treated using the microwave digestion unit MARS 5 (CEM, Kamp-Lintfort, Germany). The samples were linearly heated to 170 °C within 5 min and were held for another 3 min and subsequently cooled down to room temperature. After digestion, TP was measured as ortho-phosphate (o-PO_4_^3−^) with a Shimadzu UV-2450 spectrophotometer (Tokyo, Japan) according to the European standard procedure EN ISO 6878:2004.

The permeate Ca^2+^ and Mg^2+^ concentrations were determined using a 4100 MP-AES system from Agilent (Mulgrave, Australia). Prior to routine measurement, the AES was always calibrated using ICP standard solution IV. For routine measurement, 0.5 mL of the sample was first diluted with 9.5 mL ultra-pure water and then mixed with 0.4 mL CsCl solution (50 g L^−1^). The emission of Ca^2+^ was measured at 393.366 nm and 422.673 nm. The emission of Mg^2+^ was measured at 280.271 nm and 285.213 nm.

The sulphate concentration was measured using DX-120 ion chromatography (Dionex, CA, USA) equipped with anion exchange column IonPac AS14A (PEEK, 3.0 × 150 mm, Dionex, Sunnyvale, CA, USA). Ion separation was performed using a sodium carbonate/sodium bicarbonate (0.8 M Na_2_CO_3_/0.1 M NaHCO_3_) eluent. The multicomponent standard II for anions was used for calibration (Merck, Darmstadt, Germany). All recorded data were evaluated using Peaknet software (Fisher Scientific, Waltham, MA, USA).

## 3. Results

### 3.1. Rejection and Phosphonate Concentration in Permeates under Standard Conditions

We investigated the rejection of AMPA, IDMP and ATMP during NF and RO processes. We have chosen three different water compositions, i.e., ultra-pure water, synthetic tap water with limited divalent ion concentrations and local tap water. The latter represents natural water with common ion concentrations and ratios. 

**Figure 2:**
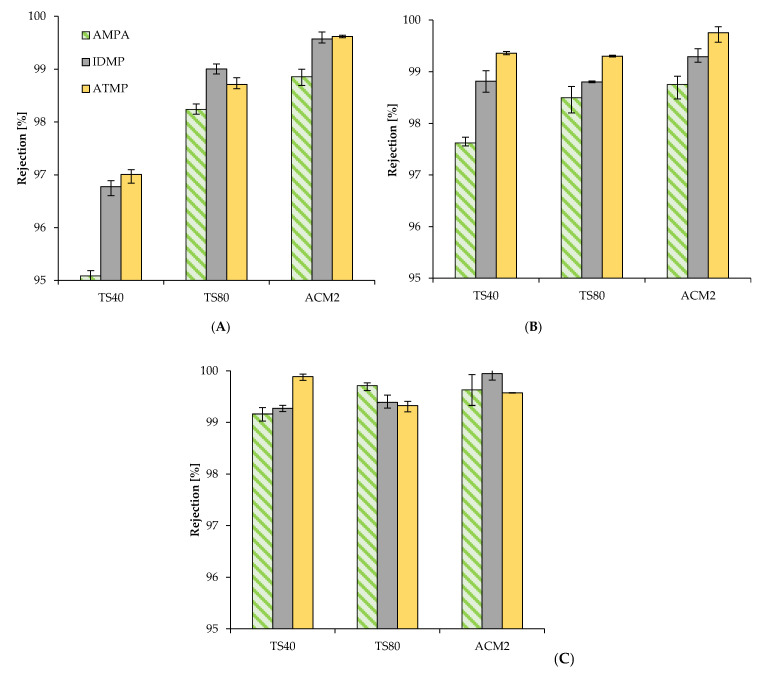
Rejection of ATMP, IDMP and AMPA with NF and RO membranes treated with three different water qualities based on TP analyses; (**A**) Ultra-pure water; (**B**) Synthetic tap water; (**C**) Local tap water; The feed concentration was 10 mg L^−1^ for all phosphonates used.

Our results clearly demonstrate that none of the investigated membranes, neither NF nor RO rejected AMPA, IDMP or ATMP completely (Figure 2A–C). Furthermore, the three phosphonates were less rejected in ultra-pure water compared with synthetic tap water and local tap water. With ultra-pure water, we determined the lowest rejection for the smallest phosphonate AMPA with all three tested membranes (Figure 2A). The AMPA rejection for the NF membrane TS40 averaged 95.1 ± 0.15%. For the NF membrane TS80 and the RO membrane ACM2, the rejection was 98.2 ± 0.09% and 98.9 ± 0.17%, respectively. Both IDMP and ATMP were better rejected with all three membranes. For IDMP and ATMP, the lowest rejection was measured with the NF membrane TS40 averaging 96.8 ± 0.11% and 97.0 ± 0.03%, respectively.

In comparison, the rejection of phosphonates in synthetic tap water with three membranes was overall higher than in ultra-pure water (Figure 2B). The lowest rejection was measured for AMPA. It averaged only 97.6 ± 0.24% for the NF membrane TS40. The rejections of the NF membrane TS80 and RO membrane ACM2 were 98.5 ± 0.26% and 98.8 ± 0.09%, respectively. IDMP and ATMP were again better rejected with all three membranes, whereas ATMP was rejected best.

In contrast to ultra-pure and synthetic tap water, the phosphonate rejection in local tap water showed no significant differences between different membranes (Figure 2C). With all three membranes, the rejection of AMPA was significantly higher than in ultra-pure and synthetic tap water (always higher than 99.0%). The IDMP rejection was similar for both NF membranes (i.e., 99.4%). The highest rejection of IDMP was achieved with the RO membrane ACM2 (99.9 ± 0.06%). Interestingly, we determined the highest ATMP rejection averaging 99.9 ± 0.01% for the NF membrane TS40, followed by 99.6 ± 0.06% for the RO membrane ACM2 and 99.3 ± 0.10% for the NF membrane TS80.

In order to verify the results from TP analyses, we also determined the phosphonate concentration in the permeates of the three different water compositions from the three different membranes (Table 2 and Table 3). In particular, our recently developed LC/MS method is powerful to identify and quantify directly the three investigated aminophosphonates under standard conditions, while the TP analysis provides a more general overview of the total phosphorus concentration. Thus, applying our LC/MS method, we were capable of strengthening our TP measurements.

For the analyses with ultra-pure water, we were capable of applying both TP and LC/MS analyses (Table 2). Overall, we always found higher phosphonate concentrations determined by TP analyses compared with LC/MS analyses. This might be due to the different analytical instrumentations and approaches applied. However, the difference between TP and LC/MS analyses indicates systematic differences. Therefore, we considered both approaches.

In ultra-pure water, we determined AMPA in all three permeates. The highest AMPA concentration occurred in the permeate of the NF membrane TS40 averaging 205.1 ± 3.9 µg L^−1^ based on LC/MS analyses. We determined IDMP in permeates of the NF membranes TS40 and TS80 averaging 192.2 ± 18.8 µg L^−1^ and 44.3 ± 6.8 µg L^−1^, respectively. IDMP was not detectable in the permeate of the RO membrane ACM2. ATMP was only detected in permeates of the NF membrane TS40 averaging 184.0 ± 16.9 µg L^−1^. In permeates of the membranes TS80 and ACM2, ATMP was not detectable by LC/MS analyses.

For the permeate samples obtained from synthetic tap water and local tap water, we were not capable of performing LC/MS analyses due to the increased concentration of cations and anions that precipitated during the evaporation. For a better demonstration of salt precipitation, we evaporated the permeates of synthetic tap water and local tap water of the three different membranes in glass instead of plastic ware (Figure S3). Unfortunately, these strong precipitations inhibited further LC/MS analyses of these samples. For that reason, we were only capable of providing results based on TP analyses for synthetic tap water and local tap water. According to TP analyses, the phosphonate concentrations in the permeates of synthetic tap water were comparable to those measured in ultra-pure water (Table 3). The resulting phosphonate concentration in the permeates seemed to decrease with increasing molecular size. Determination of optimal work pressure based on conductivity for the three tested membranes was shown in Figure S4. More in detail, we again determined the highest permeate concentrations for AMPA with all three membranes. However, in permeate of the NF membrane TS40 (234.1 ± 25.1 µg L^−1^), we measured almost doubled concentrations compared with the permeate of the RO membrane ACM2 (126.2 ± 9.1 µg L^−1^). For IDMP, we found a different trend. The IDMP concentration in the permeates was almost identical for both NF membranes TS40 and TS80 and lowest for the permeate of the RO membrane, ACM2. A similar trend was indicated for the concentration of ATMP. Both permeates of the NF membranes showed almost identical concentrations, and the permeate of RO membrane ACM2 has had the lowest ATMP concentration averaging only 22.4 ± 2.6 µg L^−1^.

Interestingly, we could not find similar relation for the three permeates if local tap water was used (Table 3). With local tap water, we found the highest AMPA concentration in the permeate of the NF membrane TS40 and the lowest for the NF membrane TS80, averaging 27.7 ± 7.8 µg L^−1^. For IDMP, the concentration in the permeates of both NF membranes was not very different. We determined the lowest IDMP concentration in the permeate of the RO membrane ACM2 averaging 39.9 ± 7.2 µg L^−1^. Surprisingly, the lowest ATMP concentration was measured in the permeate of the NF membrane TS40 averaging 11.5 ± 0.01 µg L^−1^ and the highest concentration in the permeate of the membrane TS80 averaging 67.1 ± 8.3 µg L^−1^. The ATMP concentration in the permeate of the RO membrane ACM2 was four times higher compared with the concentration in the permeate of TS40.

### 3.2. Ion Rejection with and without Phosphonates

Complexation and/or other interactions of divalent cations, such as Ca^2+^ and Mg^2+^, with the three phosphonates might also influence the rejection behaviour of the three membranes tested. In contrast to Ca^2+^ and Mg^2+^, the interaction between sulphate (SO_4_^2−^) and phosphonates has not been expected. This divalent anion commonly shows very high rejection in NF. Therefore, we assumed different rejection behaviour of sulphate compared to the other two ions Ca^2+^ and Mg^2+^.

**Figure 3:**
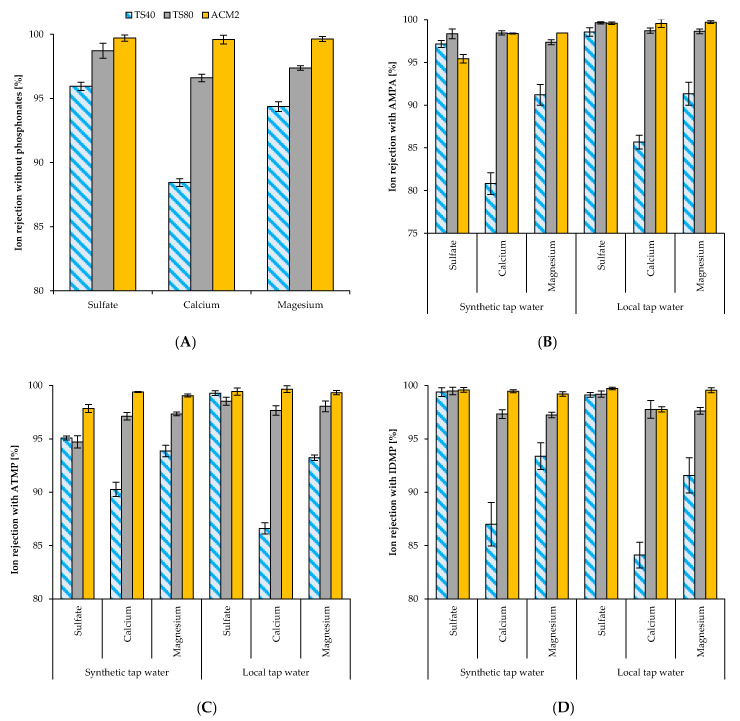
Divalent ion rejections with and without the addition of phosphonate; (**A**) Reference rejection results of tested membranes without phosphonate addition; (**B**) Rejection of divalent ions with 10 mg L^−1^ AMPA addition; (**C**) Rejection of divalent ions with 10 mg L^−1^ IDMP addition; (**D**) Rejection of divalent ions with 10 mg L^−1^ ATMP addition.

For better comparison, we first carried out ion rejection with the three membranes under optimal test conditions according to the manufacture datasheets. These filtration experiments were performed without phosphonate addition (Figure 3A). Thus, the presented results of the reference ion rejection tests show the rejection behaviour independent of possible ion-to-phosphonate interactions. Overall, we determined the lowest ion rejection with the NF membrane TS40 and the highest rejection with RO membrane ACM2 (Figure 3A). The rejection of SO_4_^2−^ was highest with all three membranes ranging between 95.9% and 99.7%. The Mg^2+^ rejection was almost similar for all membranes, ranging between 94.4% and 99.6%. The Ca^2+^ rejection was lowest for all three membranes ranging between 88.4% and 99.6%.

The ion rejection of both synthetic tap water and local tap water was slightly influenced by AMPA addition (Figure 3B). The SO_4_^2−^ rejection with NF membranes ranged between 98.5% and 99.6% independently from AMPA addition. SO_4_^2−^ rejection with the RO membrane ACM2 decreased from 99.6% to 95.8%. The influence of AMPA addition was even more obvious for Ca^2+^ and Mg^2+^ rejection. The Ca^2+^ rejection of the NF membrane TS40 decreased with both synthetic tap water and local tap water to 80.9% and 85.8%, respectively. NF membrane TS80 and RO membrane ACM2 seemed to be less affected by the addition of AMPA with regard to the Ca^2+^ rejection. For Mg^2+^, we determined a similar trend. The rejection in synthetic tap water decreased to 91.1%, while that in local tap water was 91.3%. The influence of AMPA addition on the Mg^2+^ rejection with the NF membrane TS80 and RO membrane ACM2 was negligible. Determination of optimal flux based on ion rejection for the three tested membranes was depicted in Figure S5. 

In contrast to AMPA, the addition of IDMP seemed to improve the rejection of SO_4_^2−^ for both NF membranes and with synthetic tap water and local tap water (Figure 3C). No effects were observed for the SO_4_^2−^ rejection with RO membrane ACM2. The Ca^2+^ rejection seemed to be only affected with the NF membrane TS40 in local tap water, where the rejection decreased slightly compared with the reference, from 88.4% to 84.1%, respectively. For the NF membrane TS80 and RO membrane ACM2, the IDMP addition seemed to have no negative effect on the Ca^2+^ rejection. We also determined no significant effect of IDMP addition to the Mg^2+^ rejection. Finally, we compared the effect of ATMP addition to the rejection of the three divalent ions with the three membranes (Figure 3D). Interestingly, the addition of ATMP seemed to have different effects on the SO_4_^2−^ rejection with synthetic and local tap water. With synthetic tap water, we determined slight decreases for the NF membrane TS80 and RO membrane ACM2 while the rejection with the NF membrane TS40 remained unaffected. In local tap water, the SO_4_^2−^ rejection with NF membrane TS80 and RO membrane ACM2 remained unaffected high. However, the rejection with local tap water and NF membrane TS40 increased from 95.9% in the reference experiment to 99.3%. The rejection of Ca^2+^ was slightly improved in synthetic tap water for both NF membranes and remained unaffected for the RO membrane. In local tap water, we determined only with NF membrane TS40 a slight decrease from 88.4% Ca^2+^ rejection in reference to 86.5%. The Mg^2+^ rejection seemed to be unaffected with all three membranes and both water compositions.

### 3.3. Rejection Phosphonate of Technical Formulation Containing Antiscalant

Finally, we tested our three membranes with a commercial antiscalant product containing ATMP as the main phosphonate source. In order to obtain real treatment conditions, we used local tap water for the filtration tests. Unfortunately, this further impeded LC/MS analyses but allowed at least TP analyses in both permeates and rejected feed solutions.

**Figure 4:**
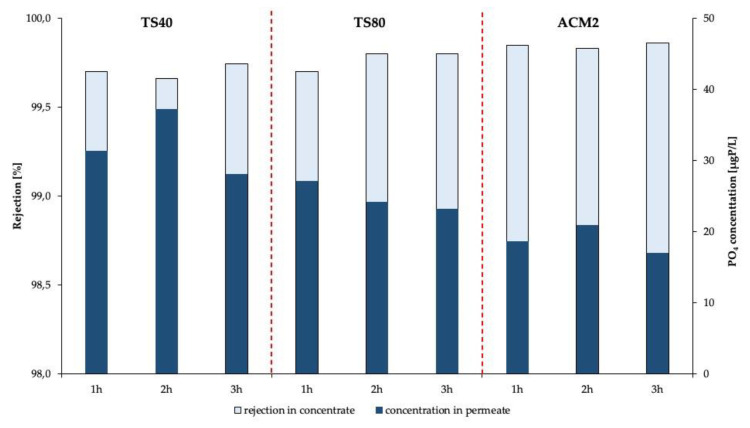
The determination of phosphate traces of antiscalant in membrane permeates of NF membranes TS40 and TS80 and RO membrane ACM2. Feed concentration antiscalant 10 mgP.

We found in all permeate samples and for all three tested membranes phosphate concentrations above 15 µgP L^−1^ (Figure 4). The highest phosphate concentration was determined for the permeates of the NF membrane TS40 (between 28.0 µgP L^−1^ and 37.2 µgP L^−1^) and the lowest for the RO membrane ACM2 (between 16.9 µgP L^−1^ and 20.9 µgP L^−1^). Furthermore, our results demonstrate that the possible rejection decrease was not due to time-dependent sampling within the three hours sampling campaign. Thus, our triplicates are justified for the three permeate concentration averaging for NF membrane TS40 a phosphate concentration of 32.2 ± 4.6 µgP L^−1^. The mean concentrations for the NF membrane TS 80 and the RO membrane ACM2 were 24.8 ± 2.0 µgP L^−1^ and 18.8 ± 1.9 µgP L^−1^, respectively. The rejection of phosphonates followed a similar trend. Determination of optimal work pressure based on ion rejection for the three tested membranes was shown in Figure S6. We determined the lowest rejection for the NF membrane TS40, averaging 99.7 ± 0.04%, followed by NF membrane TS80 averaging 99.8 ± 0.06%. The highest rejection was measured with RO membrane ACM2 averaging 99.9 ± 0.02%.

## 4. Discussion

### 4.1. Influence of Size Exclusion and MWCO during NF and RO with Phosphonates

We have investigated the potential permeability of NF and RO membranes for the aminophosphonate ATMP that is a common constituent of antiscalants. IDMP and AMPA were included in our study due to their potential of being metabolites of ATMP. We have chosen the three different water qualities UPW, synthetic tap water and local tap water, providing different ion compositions. Our UPW did not contain cations and anions; therefore, we can exclude ion interaction of our three tested aminophosphonates, especially with cations. Thus, the possible permeability of our three tested aminophosphonates can be attributed only to their individual molecular size and chemical behaviour with the membranes. Our synthetic tap water contained only a minimal cation and anion concentration compared with our local tap water. Our synthetic tap water was only composed of calcium and magnesium as major cations to better study the influence of both interactions with the three aminophosphonates and the membranes. Finally, we applied our tap water as an applicable medium providing realistic conditions such as for drinking water treatment.

Our results demonstrated that all three aminophosphonates were detectable in all investigated permeates at trace levels. As expected, we found the lowest rejection of our three aminophosphonates with UPW, followed by synthetic and local tap water. Therefore, we can conclude that the molecular size of our three aminophosphonates indeed played an important factor with regard to their membrane permeability. However, as mentioned above, the application of NF and RO filtration with UPW does not reflect real drinking water applications. Our results with synthetic and local tap water indicated clearly that the rejection of the three aminophosphonates seemed to depend on more than only the molecular size.

In particular, in microfiltration and ultrafiltration processes, the pore size and the molecular weight (size sieving) are major parameters for successful particle separation. The molecular size of the solute has a major influence on the separation behaviour of NF membranes [21]. In RO processes, only the size of ions such as hydronium and their charge can affect the membrane performance, while larger molecules such as the studied phosphonates are commonly rejected [22]. It might be for that reason that the smallest phosphonate, AMPA, was in most cases less rejected with the NF membranes TS40 and TS80 than the larger phosphonates IDMP and ATMP. These findings might be correct, at least for the filtration with UPW and synthetic tap water. For our local tap water, however, we found a similar relationship between molecular size and permeability of phosphonates only for the NF membrane TS40. For the NF membrane TS80 and RO membrane ACM2, we could not identify such a relationship. Further, we conclude that size exclusion was not the only driving parameter influencing the different rejections of the three phosphonates.

Besides sieving effects through molecular size exclusion, Kovács and Samhaber [21] pointed out that molecular size as a sole parameter ignores the shape of the permeating molecule and delivers only a rough estimation. For that reason, it is recommended to use the MWCO indicating the molecular weight of hypothetical solute that is 90% rejected [21]. Both NF membranes applied in our experimental set-up had different MWCO, i.e., 200–300 Da for the NF membrane TS40 and 100–200 Da for the NF membrane TS80, respectively. The molecular weight of AMPA is 111 g mol^−1^. IDMP has a molecular weight of 205 g mol^−1^, and ATMP has 299 g mol^−1^. Thus, all three phosphonates are in the range of 100–300 Da. Therefore, the lower rejection for the NF membrane TS40 might also be justified. Consequently, we can conclude that both molecular size and MWCO are important parameters attributing to the rejection of the three phosphonates but not exclusively.

### 4.2. Influence of Physico-Chemical Properties during NF and RO with Phosphonates

Recent literature reported that in NF and RO processes, physico-chemical properties mainly influence the separation mechanisms and/or behaviour. Different mechanisms such as size exclusion [23], solution-diffusion mechanisms [24], charge interactions [25], adsorption and concentration polarization phenomena [26] have been proposed. Furthermore, many authors stress the fact that the separation mechanism cannot be generalised and are often very complex combinations of several effects in parallel [27]. In view of this, we also have to assume that the different rejection rates found for our three aminophosphonates cannot be easily explained by one or two phenomena commonly occurring in NF and RO processes. Consequently, we have to assume that our NF experiments were strongly affected by various parameters and/or membrane properties, such as the charge density of the membrane (Donnan exclusion) [28,29], feed ionic strength [30], feed concentration [31], feed pH [32], electrostatic interactions [33] and dielectric exclusion [34,35], resulting in hindered convective and diffuse transport forces. Similar effects were also described to have affected the rejection behaviour of RO membranes, e.g., feed pH [36], ion strength [37] and Donnan exclusion [38]. Therefore, we also have to consider that those effects had a strong influence on the separation mechanism during our RO filtration experiments.

As above-mentioned, we had to reconsider several influences. Consequently, we assume that the Donnan exclusion had a major impact on the membrane properties during our filtration experiments for both NF and RO. The dissociation of functional groups at neutral pH often results in negative charges of the polymer chains of the membrane, which cause additional electrostatic interactions with the solvent [19]. This is well described in recent literature for polyamide membrane materials, making them also more hydrophilic [32]. In case the membrane interface is negatively charged, different interactions with phosphonates are conceivable. In particular, AMPA carries just one phosphonic acid group, while IDMP and ATMP carry two and three phosphonic acid groups. Thereby, the negative net charge of ATMP is considerably higher than that of IDMP and AMPA. We can expect more repulsion between ATMP and the membrane surface, resulting in higher rejection compared with IDMP and AMPA. As a result, AMPA rejection is lower than ATMP and IDMP. We found a good correlation for this hypothesis with the NF membrane TS40 but not for the NF membrane TS80. This difference between both NF membranes might indicate different separation mechanisms. However, the negative net charge of phosphonates alone does not allow such a conclusion. Other impacts such as the feed pH have to be included in this context.

The feed pH strongly affects the membrane charge and then the selectivity and rejection. The influence of the pH sensitivity of NF and RO membranes was intensively investigated and demonstrated [36,37,38]. Cancino-Madariaga et al. [36] showed that the rejection of ammonium increased with higher pH for both NF and RO membranes. Bandini et al. [38] also verified pH effects on monovalent ion rejection on polyamide NF membranes. Nowadays, the amphoteric behaviour for a wide class of polymeric membranes is well known [39]. These hydrophilic amphoteric polymers such as our polyamide membranes TS80 and ACM2 possess dissociable carboxylic and amino groups exhibiting negative or positive surface charge depending on pH [40,41]. Most NF and RO membranes have their isoelectric point below neutral pH, i.e., in a range between pH 3 and 6. Above this range, a negative net charge occurs on the membrane surface [42,43]. With regard to our results, we can expect that the different feed pH strongly affected the separation behaviour of the three membranes and the rejection of phosphonates. More in detail, our ultra-pure water with solubilised aminophosphonates had different resulting pH values. The AMPA solution achieved pH 4.5, while pH 4.0 and pH 3.6 were achieved for IDMP and ATMP, respectively. We conclude that the three different pH values of the three phosphonates affected the separation mechanism of the membranes differently by influencing their surface charge with different intensities. This was most obvious for the filtration experiments of NF membrane TS40.

Our synthetic tap water had a constant pH value of 6.5 for all three phosphonates, and the local tap water also had a constant pH value of 7.2 for all three phosphonates. The different pH values had at least two important effects on our filtration experiments. Thus, the effect of the pH of our synthetic and local tap water might result in similar effects because they are very close.

On the one hand, different pH values led to different protonation of the amino groups of the phosphonate and different deprotonation (single or double) of the acidic phosphonate moiety. Thus, our ultra-pure water might mainly promote protonation of the three phosphonates, while our synthetic tap water and our local tap water mainly promote deprotonation. On the other hand, different pH values also led to different net charges of the membrane surface. Consequently, both protonation/deprotonation of the aminophosphonates and the resulting membrane net charge have major effects on the separation mechanism, and following, on the rejection of the phosphonates with the three membranes used. We, further, assume that lower rejection for ultra-pure water was caused by minimum interaction around the isoelectric point of the membranes. Conversely, the rejection increases with increasing pH, as demonstrated with our synthetic tap water and local tap water. Therefore, we always found the highest phosphonate rejection with local tap water. This was independent of the membrane used because the pH influences the separation of both NF and RO membranes.

### 4.3. Influence on Divalent Ions Rejection in Presence of Phosphonates

Our three different water compositions not only provided different pH values but also different water compositions, i.e., different species and concentrations of monovalent and divalent ions, resulting in different feed ionic strength (conductivities). Commonly, NF membranes have a higher rejection of divalent ions than monovalent ones [44]. For RO membranes, ion rejection can differ depending on the salt composition (single or multivalent salt solution) [22,39]. The rejection of inorganic salts is mainly driven by electrostatic interactions and Donnan exclusion [31].

Since ion rejection is a primary aim of NF and RO, we also focussed our attention on the rejection of the three divalent ions SO_4_^2−^, Ca^2+^ and Mg^2+^ and their possible interactions with our three aminophosphonates. Again, we cannot generalise conclusions concerning the rejection behaviour and/or interaction with phosphonates for all three investigated divalent ions with the three membranes. Our results indicated in some cases improving effects through the addition of phosphonates and, in some cases, also declining effects.

More in detail, we conclude that the SO_4_^2−^ rejection was in most cases improved by the addition of phosphonates, especially with our local tap water. The lower rejection with our synthetic tap water might be caused by the partial protonation of SO_4_^2−^ ions leading to a formation of HSO_4_^-^ as recently reported by [22] for RO membranes. HSO_4_^−^ ions permeate through both NF and RO membranes and lead to lower SO_4_^2−^ rejection.

Our results also confirmed that the divalent ions Ca^2+^ and Mg^2+^ were generally less rejected than SO_4_^2−^. This was also true for the reference rejection test without aminophosphonates. The addition of ATMP showed, as expected, slight improving effects on the Ca^2+^ rejection, while Mg^2+^ rejection was, in most cases, unaffected. These results are consistent with our expectation since ATMP is commonly applied to predominantly prevent the crystallisation process of Ca^2+^. The addition of AMPA and IDMP showed in most cases no improvement or declining Ca^2+^ rejection because both potential metabolites of ATMP showed lower supersaturation capability than ATMP for Ca^2+^ and Mg^2+^. Nevertheless, the slight decrease in Ca^2+^ rejection with our local tap water with the NF membrane TS40 might be a result of still unknown interactions between Ca^2+^ and IDMP.

With regard to further possible interactions between the cations, ATMP and the membranes, the pH ought to gradually affect only their rejection. However, our results indicated higher divalent ion rejection at higher pH values. This finding is consistent with those in recent literature reported for RO processes [38,45]. The rejection of divalent ions such as Ca^2+^ was basically dependent on the ion concentration and presence of multivalent ions, i.e., other divalent and monovalent ions. Apart from NF membrane TS40, we mainly found higher Ca^2+^ and Mg^2+^ rejection with our local tap water for NF membrane TS80 and RO membrane ACM2. Since the influence of the pH for these divalent ions might be less important on their rejection, we have to assume that the presence of other monovalent and divalent ions and possible interactions with phosphonates positively affect their rejection.

It is also worth mentioning that the overall aminophosphonate rejection was always higher compared to the rejection of Ca^2+^ and Mg^2+^. This might also be due to the negative net charge of the membranes and the phosphonates resulting in repulsive effects. Those solute–solute interactions also have great importance on their individual rejection and separation mechanism [27,46]. The impact of divalent ions interacting with the phosphonates ought to be not underestimated. Studnik et al. [3] reported that one molecule such as ethylenediaminetetra(methylenephosphonic acid) (EDTMP) could retain 5000–10,000 Ca^2+^ molecules in solution. Thus, ATMP could also retain between 3500 and 6000 Ca^2+^ molecules in solution. Certainly, these approximations require specific experimental conditions. Nevertheless, Greenlee et al. [46] found that the addition of antiscalants such as ATMP affected the size distribution, particle morphology and calcium carbonate phases formed during the RO process. Thus, the interaction between phosphonates and divalent ions such as Ca^2+^ and Mg^2+^ affect also the permeate flux and have to be considered as a major effect during the filtration process in NF and RO. Therefore, we conclude that the rejection behaviour of the membranes tested in this study is not only strongly affected by physico-chemical properties but also by solute–solute interactions, i.e., aminophosphonate–ion. Certainly, more investigations have to be carried out to better understand their complex nature and relationship influencing the separation mechanism and rejection behaviour of the membranes with respect to aminophosphonates.

### 4.4. Technical Antiscalant Formulation in Local Tap Water Permeate

In order to verify our results obtained with different water compositions and single phosphonate treatments, we used the same experimental set-up with a technical formulation containing the antiscalant ATMP. Our results confirmed that at least traces of phosphorus were detectable in all membrane permeates. Similar to our previous results, the highest phosphorus concentration was measured in the permeate of NF membrane TS40 and the lowest in the permeate of RO membrane ACM2. Thus, it can be concluded that similar trends occurred for investigations with ATMP under standard conditions and also with the technical formulation of a commercial antiscalant product.

Our analytic equipment allowed only TP measurements of the permeates due to high cation ion impurities (Figure S3), impeding the LC/MS analysis. The high level of cation precipitation during evaporating the collected permeate samples required specific sample treatment that, unfortunately, was not combinable with our LC/MS analysis for aminophosphonates. Therefore, we were not able to determine the resulting aminophosphonate concentration but the TP concentrations. According to the safety data sheet of the technical formulation, the applied antiscalant contained not only ATMP but also hydroxyethelidene(diphosphonic acid) (HEDP). Both phosphonates have a concentration range from 5% to 15%. This makes the determination of the resulting phosphonate concentration in concentrates and permeates based on TP measurements quite difficult. Therefore, comparing our results obtained from our local tap water experiments with ATMP and those from the commercial antiscalant containing ATMP are not permissible. In spite of this, we are aware that modern drinking water treatment membrane permeates are often blends of treated and untreated waters, maintaining a certain ion strength (i.e., conductivity). For that reason, we assume that in drinking water applications, the resulting phosphonate concentration ought to be much lower, as determined in our presented study. In real drinking water applications, we estimated a concentration at a very low trace level below 10 µg L^−1^.

Recently, Armbruster et al. [6] reported the determination of phosphorus impurities of phosphonates in permeates of drinking water treatment plants during the RO process. In their extensive study, they showed up to 20% phosphorus impurities that contributed to ATMP as their original phosphonate source. They emphasized the fact that the determination of antiscalant product purity is nontrivial and requires sophisticated analytical methods. In fact, with our analytical methods, we were not able to distinguish between nominal compounds and impurities of the antiscalant. For that reason, we cannot exclude that our permeates also contained a certain portion of phosphonate impurities. Independent of this, we found similar rejection trends of the technical formulation as compared with the single phosphonate as discussed above. Therefore, we assume that individual phosphonates had similar rejection behaviour to the technical formulation. However, this is a very important aspect to be integrated into an advanced experimental set-up for future research.

## 5. Conclusions

Traces of aminophosphonates were determined in all investigated permeates independent of the water composition. We identified different parameters and membrane properties strongly affecting the rejection properties and separation mechanisms of the membranes and rejection as recently reported in the literature. In particular, we conclude that size exclusion and sieving play an important role predominantly for NF membrane TS40 that has the highest MWCO. According to our presented results, size exclusion of aminophosphonates does not significantly affect the rejection of NF membrane TS80 and RO membrane ACM2.

Further, we concluded that different separation mechanisms are a result of very complex interactions between physico-chemical properties such as Donnan exclusion, feed pH, feed ionic strength and feed concentration. Solute–solute interactions, i.e., between divalent ions and phosphonates, also play an important role in aminophosphonate rejection. This aspect is still poorly understood and should be addressed in future work.

We also conducted filtration experiments with a technical formulation with all three membranes. Our results indicate similar trends with the technical formulation as compared with our standard phosphonates. We, therefore, conclude that the phosphonates of the technical formulation also permeated through the membranes at trace levels. Since drinking water treated with NF and/or RO are common blends, we estimated a resulting phosphonate concentration below 10 µg L^−1^ in real drinking water applications. However, the presence of ATMP, IDMP and AMPA in drinking water permeates is, therefore, very likely.

## Figures and Tables

**Table 1:** Comparative features of the utilised membranes.

Feature	TS40	TS80	ACM2
Application	Demineralisation and concentrate organic solutes	Water softening, high rejection of salts and uncharged organic solutes	Standard high rejection
Membrane material	Thin film, piperazine	Thin film, polyamide	Thin film, polyamide
Active membrane area (cm^2^)	84	84	84
Backing material	Non-woven polyester	Non-woven polyester	Non-woven polyester
Thickness (µm)	130–170	130–170	130–170
Feed spacer height (mil) *	44	44	44
pH range	1.0–12.0	1.0–12.0	1.0–12.0
MWCO (Da)	200–300	100–200	-
NaCl rejection (%)	40.0	80.0	99.5
MgSO_4_ rejection (%)	98.5	98.5	n.a. *
Applied pressure (bar)	9.0	9.0	15.0
Operational flux (L h^−1^ m^−2^)	34.6–51.8	41.8–69.2	41.8–60.5

* 1 mil corresponds to 25.4 µm.

**Table 2:** Determined phosphonates in permeates with ultra-pure water based on TP and LC/MS analyses (µg L^−1^).

Membrane	AMPA		IDMP		ATMP	
	TP	LC/MS	TP	LC/MS	TP	LC/MS
TS40	512.9 ± 29.8	205.1 ± 3.9	339.9 ± 4.7	192.2 ± 18.8	318.6 ± 13.3	184.0 ± 16.9
TS80	185.0 ± 8.8	191.0 ± 15.3	104.3 ± 8.2	44.3 ± 6.8	136.0 ± 12.6	n.d.*
ACM2	116.8 ± 18.3	102.3 ± 14.5	39.6 ± 11.9	n.d.	38.1 ± 13.4	n.d.

* n.d.—not detectable.

**Table 3:** Determined phosphonate concentrations in different permeates based on TP analyses (µg L^−1^).

	Synthetic Tap Water			Local tap Water		
Membrane	AMPA	IDMP	ATMP	AMPA	IDMP	ATMP
TS40	234.1 ± 25.1	114.0 ± 11.3	65.6 ± 14.9	80.6 ± 32.5	69.4 ± 11.5	11.5 ± 0.01
TS80	146.8 ± 27.3	115.1 ± 2.5	71.3 ± 0.01	27.7 ± 7.8	57.9 ± 11.3	67.1 ± 8.3
ACM2	126.2 ± 9.1	74.8 ± 20.0	22.4 ± 2.6	36.6 ± 12.3	39.9 ± 7.2	40.9 ± 6.4

## Data Availability

The data that support the findings of this study are available on request from the corresponding author.

## Supplementary Materials

The following are available online at https://www.mdpi.com/article/10.3390/membranes11060446/s1, Figure S1: Membrane lab scale plant LSta80-2 from Sima-tec used for all filtration experiments, Figure S2: Open membrane cell of the lab scale plant, Figure S3: Exemplary carbonate precipitations of each 250 mL evaporated permeate samples, Figure S4: Determination of optimal work pressure based on conductivity for the three tested membranes, Figure S5: Determination of optimal flux based on ion rejection for the three tested membranes, Figure S6: Determination of optimal work pressure based on ion rejection for the three tested membranes, Table S1: Rejection of monovalent and divalent ions without phosphonate addition (%).

## Abbreviations

AMPA—amino(methylenephosphonic acid) (AMPA); ATMP—aminotris(methylenephosphonic acid); HEDP—hydroxyethelidene(diphosphonic acid); EDTMP—ethylenediaminetetra(methylenephosphonic acid); IDMP—iminodi(methylenephosphonic acid); LC/MS—liquid chromatography-mass spectrometry; LC-ESI-MS—liquid chromatography-electro spray ionization-mass spectrometry; MWCO—molecular weight cut-off; NF—Nanofiltration; RO—reverse osmosis; SIM—selected ion monitoring; TP—total phosphorus; UPW—ultra pure water.
