# Supplementary material for: Aminophosphonates in Nanofiltration and Reverse Osmosis Permeates
Source: Membranes (Basel). 2021 Jun 15;11(6):446. doi: 10.3390/membranes11060446 (PMC8232610; doi:10.3390/membranes11060446)
Supplement: Supplementary file 1 [file membranes-11-00446-s001.zip › membranes-1261932-supplementary.pdf]

membranes

Supplementary Materials

Aminophosphonates in Nanofiltration and Reverse Osmosis Permeates

Ramona Kuhn [1,*], Carsten Vornholt [2], Volker Preuß [3], Isaac Mbir Bryant [4] and Marion Martienssen [5]

1 Chair of Biotechnology of Water Treatment, Brandenburg University of Technology Cottbus/Senftenberg, 03046 Cottbus, Germany
2 Chair of Water Treatment and Urban Hydraulic Engineering; Brandenburg University of Technology Cottbus/Senftenberg, 03046 Cottbus, Germany; carstenvornholt@gmx.de
3 Chair of Water Treatment and Urban Hydraulic Engineering; Brandenburg University of Technology Cottbus/Senftenberg, 03046 Cottbus, Germany; preuss@b-tu.de
4 Department of Environmental Science, University of Cape Coast, 4P48 + 72 Cape Coast, Ghana; ibryant@ucc.edu.gh
5 Chair of Biotechnology of Water Treatment, Brandenburg University of Technology Cottbus/Senftenberg, 03046 Cottbus, Germany; martiens@b-tu.de
* Correspondence: kuhnr@b-tu.de

1. Configuration of the Lab-Scale Membrane Plant

In the present work, the corresponding membrane tests were carried out with the laboratory system LSta80-2 from SIMA-tec GmbH (Figure S1). The plant can be used for ultrafiltration, nanofiltration and reverse osmosis. A pump conveys the process water from the storage tank to a flat sheet membrane cell. If necessary, a second membrane cell can be used. The configuration of the plant allows the operation of two membranes at the same time. The system operated in a cross flow mode. The water first flows through the first cell, then to the second. The pressure required for filtration is generated through a pressure control valve. For sampling feed, concentrate and permeate individual sampling point are available.

The opened membrane cell for injecting the test membrane is presented below (Figure S2). The area for the test membranes used was 84 cm². The membrane was placed on a spacer which caused a turbulent flow on the membrane surface. This mimics the conditions commonly present in spiral winding modules.

Using the DASYLab® software logger, parameters such as temperatures, conductivities, flow rates and pressures were measured in feed, concentrate and permeates. Stable temperature of 20 °C was set on the concentrate tank. This was achieved by applying a connected cryostat (unistat ango nuevo Company: Huber Offenburg, Germany) (Figure. S1.b).

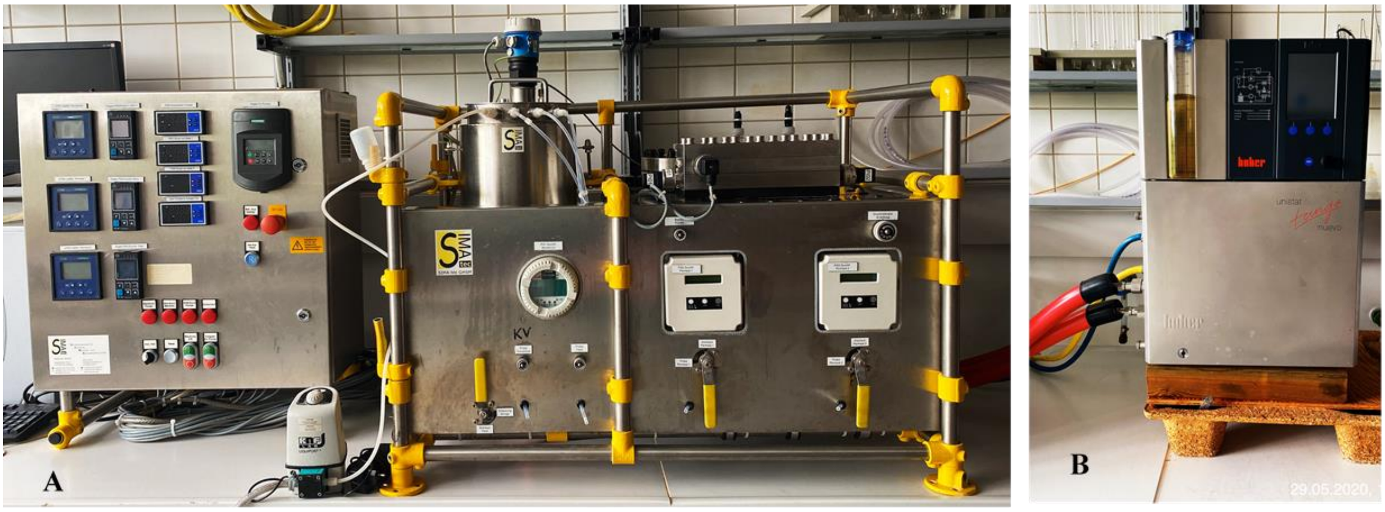

Figure S1. Membrane lab scale plant LSta80-2 from Sima-tec used for all filtration experiments. **A**: Front side. **B**: Cryostat unistat tango Nuevo.

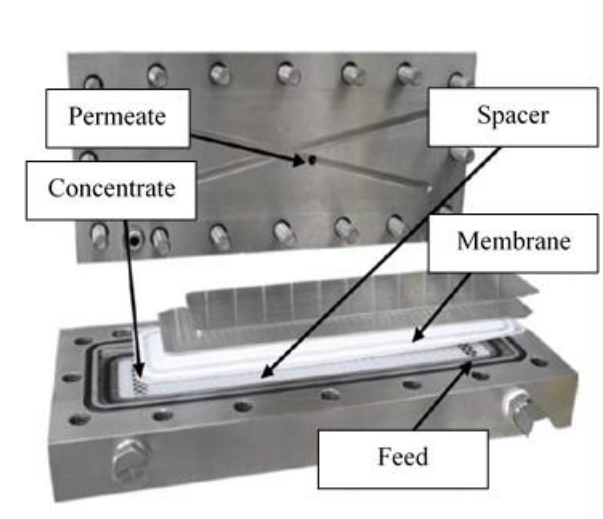

Figure S2. Open membrane cell of the lab scale plant.

2. Additional Information on Exemplary Carbonate Precipitation

The permeate samples (250 mL) obtained from synthetic tap water and local tap water were usually evaporated in plastic ware in order to avoid aminophosphonate adsorption to the walls of the beaker. However, carbonate precipitations were visible for our synthetic tap water and local tap water. We, again, carried out the evaporation in glass ware to better demonstrate the precipitation (Figure. S3). We found the lowest level of carbonate precipitation in the RO permeates for both synthetic tap water and local tap water. We detected the highest level of carbonate precipitation in the evaporated permeates of the NF membrane TS40.

Rinsing the glass wares with little volumes of ultra-pure water did not successfully resuspend the precipitates including aminophosphantes. We repeated the resuspension attempts with acid solution containing 0.1 mol HCl. Those samples were compatible with our TP analysis but not with our LC/MS analysis which requires an additional sample clean-up with cation exchange resin Dowex 50WX8. This clean-up procedure was not very suitable for AMPA due to strong AMPA adsorption to the exchange resin and low recovery rates. Following aminophosphonate quantification and mass balances would be misleading [1,2]. Therefore, under standardised condition, measurements of total phosphorus is an adequate solution.

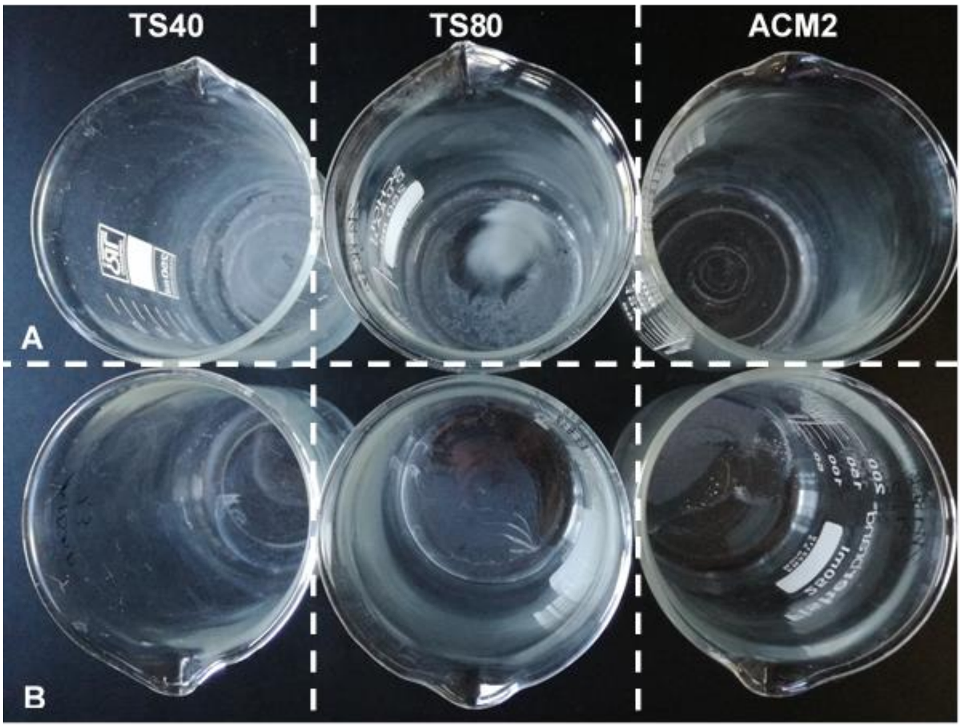

Figure S3. Exemplary carbonate precipitations of each 250 mL evaporated permeate samples. (**A**) Upper part of glasses representing evaporated local tap water permeate samples of the NF membranes TS40 and TS80 and from the RO membrane ACM2. (**B**) Lower part of glasses representing evaporated synthetic tap water permeate samples of the NF membranes TS40 and TS80 and from the RO membrane ACM2.

3. Rejection of Monovalent and Divalent Ions Without Phosphonate Addition

The ion rejection of monovalent and divalent ions is presented below (Table S1). Overall, the ion rejection was lowest for the NF membrane TS40 and highest for the RO membranes ACM2.

Table S1. Rejection of monovalent and divalent ions without phosphonate addition [%].

Membrane	Na^+	Cl^-	Ca^{2+}	Mg^{2+}	SO_4^{2-}
TS40	24.4 ± 0.84	63.7 ± 3.17	88.4 ± 1.85	94.4 ± 0.20	95.9 ± 1.03
TS80	70.6 ± 1.95	85.4 ± 1.73	96.6 ± 0.13	97.3 ± 0.12	98.6 ± 0.64
ACM2	97.7 ± 0.24	99.1 ± 0.09	99.6 ± 0.06	99.6 ± 0.05	99.7 ± 0.05

4. Influence of Other Operational Parameters During NF and RO with Antiscalants

Despite all physico-chemical parameters discussed so far, operating parameters such as operational pressure, cross-flow velocity, temperature and recovery rate also have to be taken into account as factors that affect NF and RO processes [3,4].

Prior to our investigations, we had carried out membrane screening determining optimal pressure and flux condition of all three membranes (Figure A.4 & A.5). Based on our results, we decided to operate the NF membranes at 9 bar and the RO membrane at 15

bar. In fact, increasing the operational pressure can lead to increases in concentration polarisation thus increasing the convective transport as well as solute diffusion [5]. Therefore, we did not further increase the operational pressure chosen. Furthermore, we determined optimal flux for all three membranes depending on the rejection of monovalent and divalent ions (Figure S6). We decided to operate the NF membrane TS40 with 52 L $h^{-1}m^{-2}$, which was the highest recommended operational flux according to the manufacture data sheet. Both membranes TS80 and ACM2 were operated below the operational flux range according to the manufacturer's data sheet. However, increasing the flux would not automatically lead to higher ion rejection and in addition would also lead to increase the operational pressure. Therefore, we believe that our operational parameters were best choice for subsequent test conditions with the antiscalants.

Another important fact still had to be considered. NF membranes can also be operated as low-pressure reverse osmosis (LPRO) membranes [6]. According to Chang et al. [7] they can have very similar chemical composition but different processing condition. This might be applicable to our NF membrane TS80 compared with RO membrane ACM2. Both membranes consist of the same membrane material and might have overall similar chemical composition. The flux range of the NF membrane TS80 was also very close to those of RO membrane ACM2. Thus, we could consider that the NF membrane TS80 performed the ion and phosphonate separation like a LPRO membrane rather than NF membranes with dominating ion selectivity. In other words, the separation behaviour of the NF membrane TS80 seemed to be very close to the rejection behaviour of the RO membrane ACM2, therefore, it is likely to assume that similar separation behaviour might also take place.

Finally, adsorption, fouling and scaling are very often discussed as factors that affect membrane flux and the separation mechanism during NF and RO processes [3,8,9]. We did not further investigate those phenomena in detail with our experimental set-up. However, we cannot exclude adsorption taking place and affecting the separation mechanisms during filtration especially, with NF. Since we had applied phosphonates preventing scaling, we assumed that scaling was an under-represented influence during our study. Fouling effects, especially biofouling, were considered as negligible due to the short-term performance time of our experiments. So far, we could not determine effects caused by adsorption, scaling and/or fouling negatively affecting rejection and/or flux. Nevertheless, effects such as adsorption, scaling and fouling with regard to their interaction with phosphonates should be addressed in future work.

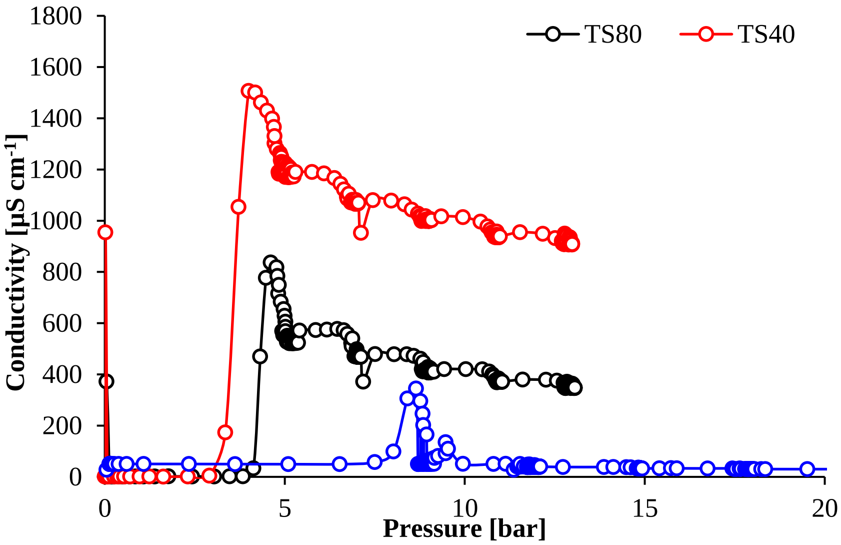

Figure S4. Determination of optimal work pressure based on conductivity for the three tested membranes.

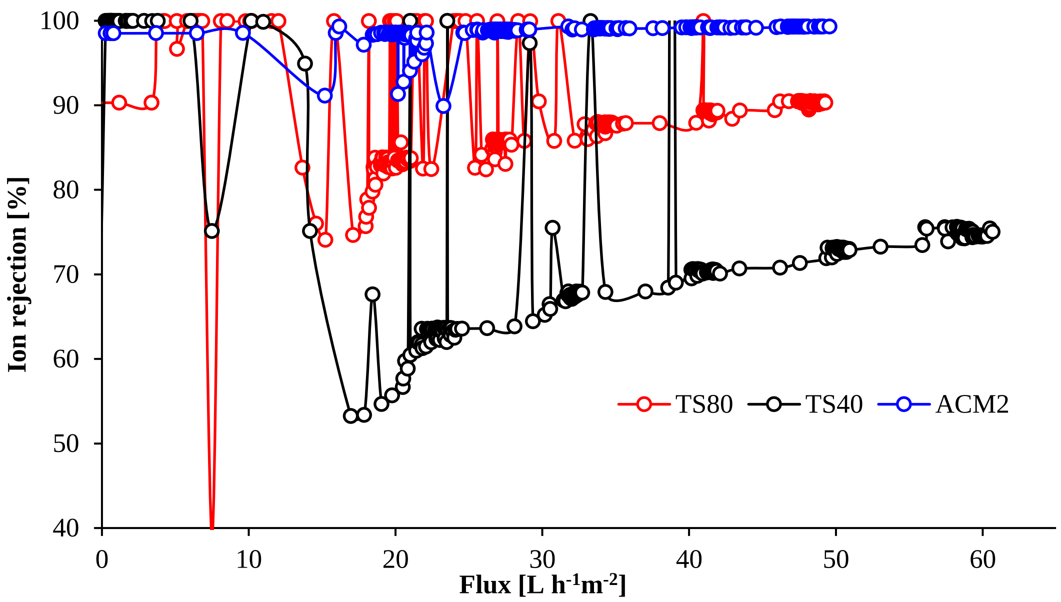

Figure S5. Determination of optimal flux based on ion rejection for the three tested membranes.

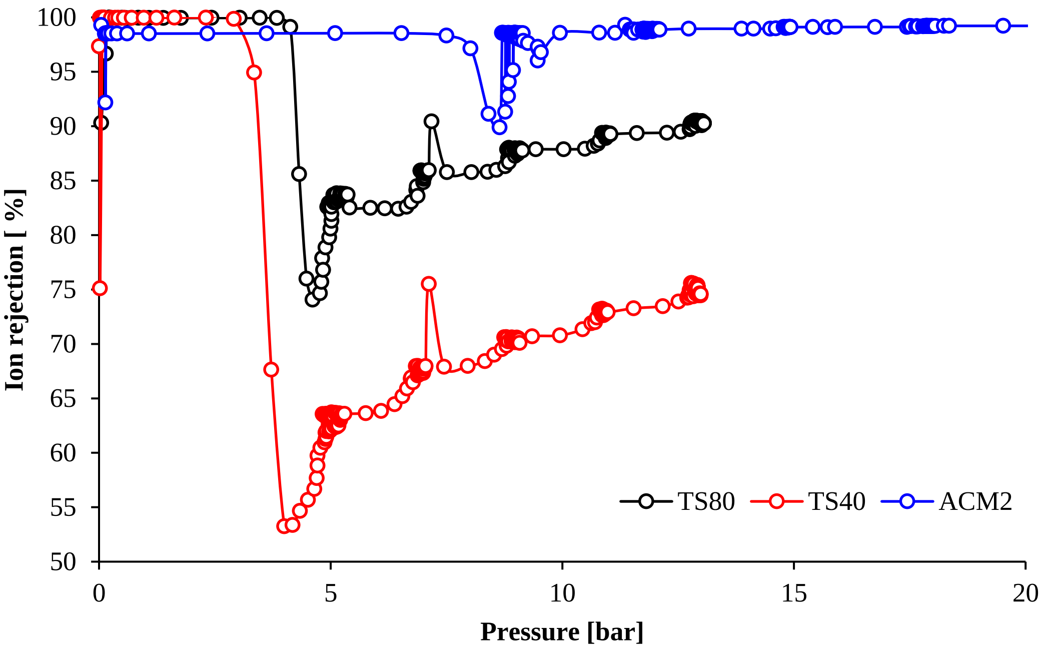

Figure S6. Determination of optimal work pressure based on ion rejection for the three tested membranes.

References

1. Kuhn, R.; Jensch, R.; Bryant, I.M.; Fischer, T.; Liebsch, S.; Martienssen, M. Rapid sample clean-up procedure for aminophosphonate determination by LC/MS analysis. *Talanta* **2020**, *208*, 120454, doi:10.1016/j.talanta.2019.120454.
2. Kuhn, R.; Bryant, I.M.; Martienssen, M. Supplementary data on rapid sample clean-up procedure of aminophosphonates for LC/MS analysis. *MethodsX* **2020**, *7*, 100933, doi:10.1016/j.mex.2020.100933.
3. Shen, J.; Schaefer, A. Removal of fluoride and uranium by nanofiltration and reverse osmosis: A review. *Chemosphere* **2014**, *117*, 679–691, doi:10.1016/j.chemosphere.2014.09.090.
4. Van Wagner, E.M.; Sagle, A.C.; Sharma, M.M.; Freeman, B.D. Effect of crossflow testing conditions, including feed pH and continuous feed filtration, on commercial reverse osmosis membrane performance. *J. Membr. Sci.* **2009**, *345*, 97–109, doi:10.1016/j.memsci.2009.08.033.

5. Han, J.; Qiu, W.; Hu, J.; Gao, W. Chemisorption of estrone in nylon microfiltration membranes: Adsorption mechanism and potential use for estrone removal from water. *Water Res.* **2012**, *46*, 873–881, doi:10.1016/j.watres.2011.11.066.
6. Ozaki, H.; Sharma, K.; Saktaywin, W. Performance of an ultra-low-pressure reverse osmosis membrane (ULPROM) for separating heavy metal: effects of interference parameters. *Desalination* **2002**, *144*, 287–294, doi:10.1016/s0011-9164(02)00329-6.
7. Donnan, F.G. Theory of membrane equilibria and membrane potentials in the presence of non-dialysing electrolytes. A contribution to physical-chemical physiology. *J. Membr. Sci.* **1995**, *100*, 45–55, doi:10.1016/0376-7388(94)00297-c.
8. Gomes, A.; Gonçalves, I.; Pinho, M. The role of adsorption on nanofiltration of azo dyes. *J. Membr. Sci.* **2005**, *255*, 157–165, doi:10.1016/j.memsci.2005.01.031.
9. Van Der Bruggen, B.; Braeken, L.; Vandecasteele, C. Flux decline in nanofiltration due to adsorption of organic compounds. *Sep. Purif. Technol.* **2002**, *29*, 23–31, doi:10.1016/s1383-5866(01)00199-x.
